# The role of surface corrugation and tip oscillation in single-molecule manipulation with a non-contact atomic force microscope

**DOI:** 10.3762/bjnano.5.22

**Published:** 2014-02-26

**Authors:** Christian Wagner, Norman Fournier, F Stefan Tautz, Ruslan Temirov

**Affiliations:** 1 Leiden Institute of Physics, Universiteit Leiden, Niels Bohrweg 2, 2333 CA Leiden, The Netherlands; 2 Peter Grünberg Institut (PGI-3), Forschungszentrum Jülich, 52425 Jülich, Germany; 3 Jülich Aachen Research Alliance (JARA)-Fundamentals of Future Information Technology, 52425 Jülich, Germany

**Keywords:** atomic force microscopy (AFM), force-field model, 3,4,9,10-perylene-tetracarboxylic-dianhydride (PTCDA), qPlus, single-molecule manipulation

## Abstract

Scanning probe microscopy (SPM) plays an important role in the investigation of molecular adsorption. The possibility to probe the molecule–surface interaction while tuning its strength through SPM tip-induced single-molecule manipulation has particularly promising potential to yield new insights. We recently reported experiments, in which 3,4,9,10-perylene-tetracarboxylic-dianhydride (PTCDA) molecules were lifted with a qPlus-sensor and analyzed these experiments by using force-field simulations. Irrespective of the good agreement between the experiment and those simulations, systematic inconsistencies remained that we attribute to effects omitted from the initial model. Here we develop a more realistic simulation of single-molecule manipulation by non-contact AFM that includes the atomic surface corrugation, the tip elasticity, and the tip oscillation amplitude. In short, we simulate a full tip oscillation cycle at each step of the manipulation process and calculate the frequency shift by solving the equation of motion of the tip. The new model correctly reproduces previously unexplained key features of the experiment, and facilitates a better understanding of the mechanics of single-molecular junctions. Our simulations reveal that the surface corrugation adds a positive frequency shift to the measurement that generates an apparent repulsive force. Furthermore, we demonstrate that the scatter observed in the experimental data points is related to the sliding of the molecule across the surface.

## Introduction

The problem of the adsorption of organic molecules presents many fundamental challenges that stem mostly from the chemical complexity of organic compounds. A complex chemical structure often leads to a wide variety of different types of interactions, the interplay of which defines the behavior of such adsorption systems [1]. With the advent and consequent rapid development of scanning probe microscopy (SPM) techniques, investigations of adsorbate–surface interactions on a single-molecule level have become possible [2–18]. Especially interesting is the possibility of probing the molecule–surface interaction while tuning its strength through a well-controlled single-molecule manipulation induced by the SPM tip [6,11,19–22]. Such experiments demand special instrumentation. It has been demonstrated that the recently developed experimental setups that combine low-temperature scanning tunneling, and qPlus-based non-contact atomic force (NC-AFM) microscopes can be a potent tool when applied to studies of single-molecule manipulation [6,11,15]. The STM function facilitates the effective preparation of the experiment while the NC-AFM, operated simultaneously with the STM, is used to control the structure and to measure the forces that act in the junction during the manipulation. Although, in principle, the conductance measured with the STM could also be used to control the structure during the manipulation of a molecule, the relation between the conductance and the structure of single-molecule junctions is still not generally understood and therefore the forces that act in the junction during the manipulation provide more direct information about the conformation of the molecule.

**Figure 1:**
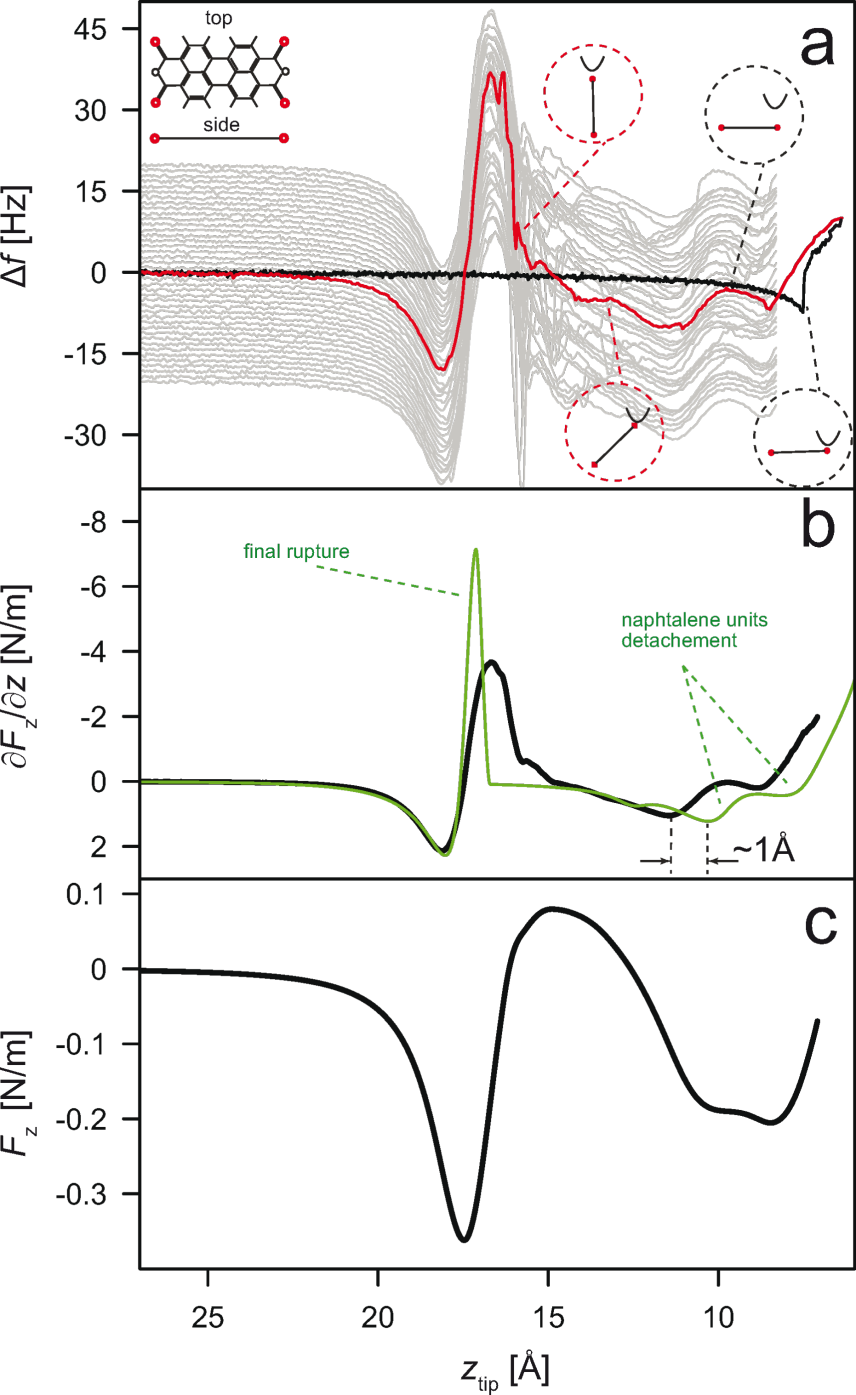
(a) Exemplary data from an experiment in which a single PTCDA molecule on the Au(111) surface was contacted, lifted up and put down again. The number of executed “lift–put” cycles is 40. The surface is located on the right. The black curve shows the initial approach and the contacting event. The first lift curve is shown in red. The consequent “lift–put” curves are shown in grey. The “lift” (“put”) curves are shifted up (down) for clarity. The inset in the upper left corner shows the chemical structure of PTCDA. (b) Generic *∂F_z_*/*∂z*(*z*) curve (black) for the lifting of PTCDA from Au(111) as obtained by averaging over seven individual contacting experiments. The green curve shows the result of simulations reported in [11]. (c) *F*_z_(*z*) force curve as obtained by direct integration of the experimental *∂F_z_*/*∂z*(*z*) shown in panel (b) in black.

One of the first attempts to manipulate large organic adsorbates with the tip of the LT-STM/NC-AFM has been made on 3,4,9,10-perylene-tetracarboxylic-dianhydride (PTCDA) molecules [6] (cf. inset of Figure 1a). This system is considered to be an archetypal case of a functional organic adsorbate [1]. PTCDA interacts with surfaces via two distinct functionalities: the π-conjugated perylene core and the carboxylic oxygen atoms attached at the four corners of the rectangular aromatic backbone. Approaching the metal tip to one of the carboxylic oxygen atoms, it is possible to form a local chemical bond between the oxygen and the outermost atom of the tip apex [23]. This bond is of sufficient mechanical strength to allow the lifting of the molecule from the surface up to the point of its complete removal. Recording the frequency shift Δ*f*(*z*) of the qPlus tuning fork during the removal of the molecule, we have previously succeeded in reconstructing the junction structure throughout the manipulation process. This has been achieved by simulating the experimental curves


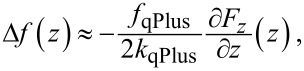


in which *f*_qPlus_ = 30.311 kHz and *k*_qPlus_ = 1800 N/m, with a custom-developed force-field model [11].

A detailed comparison between the simulation and the experiment, however, reveals systematic inconsistencies that can be attributed to three main factors that have been omitted from the initial model: i) the atomic corrugation of the surface, ii) the elasticity of the tip material, and iii) the finite amplitude of the qPlus tuning fork oscillation. Here we take a step towards more realistic force-field simulations of single-molecule manipulation by including the three factors mentioned above into the simulation model and by demonstrating that even their qualitative consideration improves the correspondence between simulations and experiment, and therefore facilitates an improved understanding of the mechanics of single-molecular junctions.

## Experimental

The details of PTCDA lifting experiments have been described previously [3,6,11]. Here we briefly repeat the essential features of the experimental procedure. We lift single PTCDA molecules (cf. inset of Figure 1a) from a Au(111) single crystal surface by using an LT-STM/NC-AFM from CREATEC [3,6,11,24] at *T* = 5 K in ultra-high vacuum. When preparing the manipulation we detach one PTCDA molecule from the edge of a molecular island with the tip and move it to a clean spot on the bare metal surface. For establishing the contact to the molecule, the tip is placed over one of its carboxylic oxygen atoms and is moved further towards the surface until a sudden increase in junction conductance and change in Δ*f* occurs (cf. Figure 1a). The conductance increases due to the snap-up of the oxygen atom to the tip, which marks the formation of a chemical tip–molecule bond [23]. Once the contact to the carboxylic oxygen atom has been formed, the tip is retracted away from the surface until the smallest distance between the surface and the atoms of the molecule suspended vertically from the tip is approximately 2 nm. After it has been removed from the surface the molecule is put back again by moving the tip towards the position at which the contact to the molecule has been initially established. In our experiments at least 30% of all tips enabled us to execute series of tens of such “lift–put” cycles without loosing the contact between tip and molecule while simultaneously recording Δ*f*(*z*) of the qPlus sensor [6,11,24].

Figure 1a, which exhibits an example of such a measurement, reveals that the qPlus sensor oscillating with the amplitude of *A*_qPlus_ = 0.2–0.3 Å can indeed measure the stiffness *∂F_z_*/*∂z*(*z*) of the junction continuously through all stages of the manipulation experiment. Since the intrinsic stiffness of the qPlus tuning fork (*k*_qPlus_) is much higher than the typical strengths of single atomic bonds, it can be used to test processes of single-bond ruptures reliably (in practice the overall success of such tests is limited by the stiffness of the metallic tip that is employed in the manipulation experiment). In particular, the superior stiffness of the qPlus sensor results in the absence of any systematic hysteresis between the stiffness curves measured in the “lift” and “put” parts of the manipulation cycle. As a result, the measurement of Δ*f* shown in Figure 1a exhibits a remarkable degree of overall reproducibility.

A closer inspection, however, reveals that in the intermediate range of tip–surface distances, 13 Å ≤ *z* ≤ 17 Å the recorded Δ*f* traces show higher scattering. Previously we avoided analyzing the detailed junction behavior in this region and concentrated exclusively on the generic features that can be clearly isolated by averaging over many individual manipulation curves (cf. Figure 1b). Averaging out the observed experimental scattering, however, does not resolve the underlying issue, as the presence of the problem becomes apparent again when we try to reconstruct the force acting in the junction by integrating the averaged *∂F_z_*/*∂z*(*z*) curve displayed in Figure 1b: In the same range, in which *∂F_z_*/*∂z*(*z*) shows higher scatter, *F_z_*(*z*) apparently becomes positive, which suggests that the molecule–surface interaction there is repulsive (cf. Figure 1c). It will be shown below that this repulsion is spurious and stems from the combined effects of surface corrugation and the finite amplitude of the qPlus oscillation. In addition, it will be demonstrated that the increased scattering of the experimental data observed in the range 13 Å ≤ *z* ≤ 17 Å can be explained by the sliding of the molecule across the corrugated surface potential. On the methodological side, the important message of this work is to demonstrate that the force-field modeling of single-molecule manipulation can be successful in explaining precise details of the NC-AFM junction mechanics. However, to do so the simulation must account for the oscillatory dynamics of the qPlus sensor.

## Simulations

We start building the force-field model of the tip–PTCDA–surface junction according to the principles that have been developed in our earlier work [11]. First we use the standard force-field approach to simulate the intramolecular mechanics of PTCDA, fitting it explicitly to DFT calculations of the mechanical properties of a gas phase molecule. The intramolecular force-field parameters are kept fixed through the rest of the simulation. The molecule–tip bond is described by a spherical Morse potential (*D* = 1.3 eV, *r*_0_ = 2.2 Å, *a* = 2.0 Å^−1^) binding one of the carboxylic oxygens to the outermost tip apex atom. The parameters of this potential have been determined with the help of DFT simulations presented in [23]. The molecule–surface interaction is described as a set of individual atom–surface potentials summed over the atoms constituting PTCDA. The surface is represented by a continuous plane that interacts with the individual atoms of PTCDA via the Pauli repulsion parameterized by an exponential potential that is proprotional to exp(−*A*_p_*z*) and the van der Waals interaction expressed as a potential proportional to *z*^−3^. We note here that the correct asymptotic behavior of the van der Waals interaction is (*z* − *z*_0_)^−3^, where *z*_0_ is the location of the van der Waals plane, usually *z*_0_ = (1/2) *d*_lattice_. However, as discussed in [24–25], this form is only valid for *z* > 5 Å while for *z* < 5 Å the van der Waals interaction is damped. We achieve this damping by letting *z*_0_ → 0. More details can be found in [24]. For simplicity it is assumed that PTCDA consists of only two types of atoms: the 26 backbone (all carbon plus the two anhydride oxygen atoms; hydrogen atom interaction is scaled by 0.25) and the four carboxylic oxygen atoms. The interaction potentials of the carboxylic oxygen and the backbone atoms are described via two separate parameter sets that amount to a total of five free parameters (if additional chemical molecule–surface interactions are absent). These five parameters, which describe the interaction of PTCDA with the surface, have been previously determined by fitting simulated *∂F_z_*/*∂z*(*z*) curves to the experiment [11] (Figure 1).

In [11] the simulation of the lifting process was carried out in the following way: The model tip was lifted perpendicular to the surface such that the *z*-coordinate of the tip *z*_tip_ increased after each step by 1 pm. At each step the molecular geometry and the lateral tip position were relaxed, the former by minimizing the net force that acts on each atom in the molecule, the latter by zeroing the lateral forces on the tip. The thus obtained *F_z_*(*z*_tip_) was numerically differentiated to obtain *∂F_z_*/*∂z*(*z*_tip_). Finally, the experimental *z*-scale was aligned to *z*_tip_ by a rigid translation of the data.

Figure 1b displays a force-gradient curve simulated as described in the previous paragraph. The comparison in Figure 1b reveals a few systematic differences between the experimental (black) and the simulated (green) curves. They, in fact, occur in the same *z*_tip_ range where Δ*f* shows higher scatter and the reconstructed experimental *F_z_* becomes repulsive. The character of the observed differences can be described as follows: First, the simulation predicts the peak in *∂F_z_*/*∂z*(*z*_tip_) that precedes the final rupture of the molecule–surface bond to be considerably sharper than the one seen in the experiment (cf. Figure 1b). Secondly, the distance between the features corresponding to the detachment of the naphthalene units of PTCDA and the final rupture of the molecule–surface bond is larger by about 1 Å in the simulation (note that in our previous analysis we had to cut the experimental curve into two pieces and align them separately with respect to the simulation because of the same problem [11]). As was mentioned above, at least some of the observed inconsistencies occur because our initial force-field model [6,11] does not reflect the actual measurement as performed with the NC-AFM. To account for this, one has to go beyond the calculation of a sequence of relaxed geometries at increasing *z*_tip_ and zeroed lateral forces. In reality lateral forces are present. This should result in the lower end of the molecule sliding through a corrugated surface potential during the lifting of the tip. The lateral displacement of the molecule over the corrugated surface will be induced by the retraction of the tip as well as by the vibration of the qPlus sensor. As a result, the amplitude of the qPlus oscillation, although small, cannot be neglected any more.

To adapt the model accordingly, we change it in several steps. First we introduce the corrugation of the surface, parameterized with a simple 2D cosine potential

**Figure 2:**
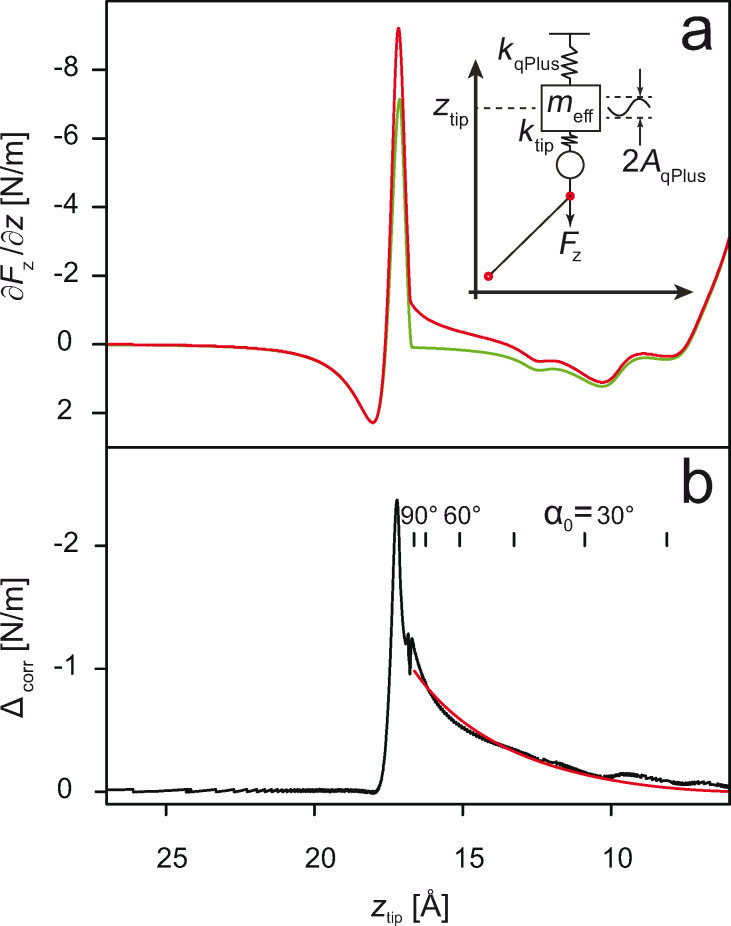
(a) Comparison between the *∂F_z_*/*∂z*(*z*) curves obtained from the initial ([11]) and the extended (this work) force-field model. The green curve is the same as the one shown in Figure 1b and was obtained with the model that accounts neither for oscillation of the qPlus sensor nor for the corrugation of the surface. The red curve is produced with the model taking both of the above effects into account (cf. text). The inset in the upper right corner clarifies the schematics of the single-molecule junction used in the extended simulation model. (b) Correction term Δ_corr_ calculated as the difference between the two curves from panel (a). The red curve was obtained from the analytic expression (Equation 2) and fitted to the simulated Δ_corr_. Additional tick-marks show the correspondence between the *α*_0_ and *z*_tip_ scales (cf. text).

[1]
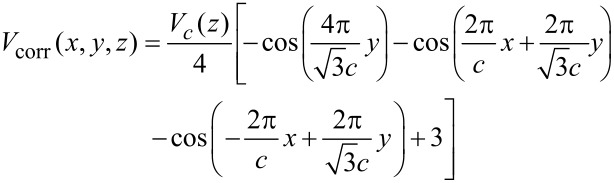


with the in-plane nearest neighbor distance *c* = 2.884 Å corresponding to the Au(111) surface structure and a corrugation amplitude *V_c_*(*z*) ≈ (2.6/*z*)^7^ that decays rapidly with increasing distance to the surface. Since here we aim at a qualitative description, the precise functional form of the corrugation potential is not relevant and we also can assume that the surface corrugation is only felt by the carboxylic oxygen atoms of PTCDA (which have the strongest tendency to form local bonds) [1]. This simplification enables a much clearer interpretation of the simulation results, in particular a direct link to an analytical model that we discuss later. Extending the model further, we allow for a finite stiffness of the tip that is simulated by introducing an additional atom situated above the tip apex atom and connected to it via a harmonic 1D potential (cf. inset Figure 2a). The stiffness *k*_tip_ of this harmonic bond is fixed, but the bond itself is allowed to relax during the simulation. In the simulation we find a maximal tip-extension of 1 Å. Assuming that a mesoscopic part of the tip relaxes, this elongation brakes up into relative atomic displacements of small fractions of an angstrom, justifying the use of an harmonic potential. Finally, the new model also accounts for the oscillation of the qPlus sensor. To do so, the complete lifting process is simulated in two stages. As before, *z*_tip_ is increased in steps of 1 pm and the structure (including the position of the lower end of the molecule as well as the extension of the tip) is relaxed. The relaxation is done by *either* allowing the lateral coordinate of the tip to change (no lateral forces in the junction are allowed, hence the lower end of the molecule does not slide over the surface) *or* fixing the lateral coordinate of the tip, thus enforcing molecular sliding if necessary. After each retraction–relaxation step the tip is moved *vertically* around *z*_tip_ in *N* = 150 steps of Δ*z* = 0.4 pm each, so that the maximum deviation (*N*Δ*z*)/4 totals to *A*_qPlus_ = 0.15 Å. At each deflection step *i* (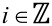, −75 ≤ *i* ≤ 75) the molecule is allowed to relax and the force *F_z_*(*z*_tip_ + *i*Δ*z*) acting on the tip from the molecule is calculated. Numerically solving the equation of motion for the qPlus sensor with the effective mass (*m*_eff_ = *k*_qPlus_/2π *f*_qPlus_ = 49.62 μg) under the influence of the total force *F_z_*_,Total_(*z*_tip_ + *i*Δ*z*) = *k*_qPlus_ *i*Δ*z* + *F_z_*(*z*_tip_ + *i*Δ*z*), we obtain the frequency of its oscillation. The time step used in the simulation of the tip oscillation is 200 ps, which corresponds to a frequency resolution of about 0.2 Hz. Note that the motion of the tip during one oscillation cycle is strictly vertical, whereas the overall motion of the tip during the retraction–relaxation steps might also involve a lateral displacement of the tip.

## Results and Discussion

To understand how the refinement of the mechanical model of the junction influences the outcome of the simulations we perform several different simulation runs with tip oscillation and surface corrugation. In the first run we make the tip infinitely stiff (*k*_tip_ = ∞) and additionally relax the lateral position of the tip after each lifting step, such that no lateral forces are present and therefore the lower end of the molecule does not slide along the surface during lifting. The resulting *∂F_z_*/*∂z*(*z*_tip_) curve is shown in red in Figure 2a. Taking the difference between the red curve and the green curve obtained in [11] with the original model, i.e., without tip oscillation and surface corrugation, we discover that the inclusion of the qPlus oscillation and the surface corrugation in the model changes *∂F_z_*/*∂z*(*z*_tip_) by adding an additional negative correction term Δ_corr_ that increases its absolute value towards the end of the manipulation (cf. Figure 2b). Note that by definition of our positive *z*-direction the force gradient *∂F_z_*/*∂z* has the opposite sign of the frequency shift. Hence, the correction term means a positive contribution to Δ*f*. If integrated, Δ_corr_ will produce an additional repulsive contribution to the force measured during the lifting, just as observed in experiment (Figure 1b and c). An understanding of the physical mechanism behind Δ_corr_ could then also clarify the unexpected appearance of the repulsive force in our measurements.

**Figure 3:**
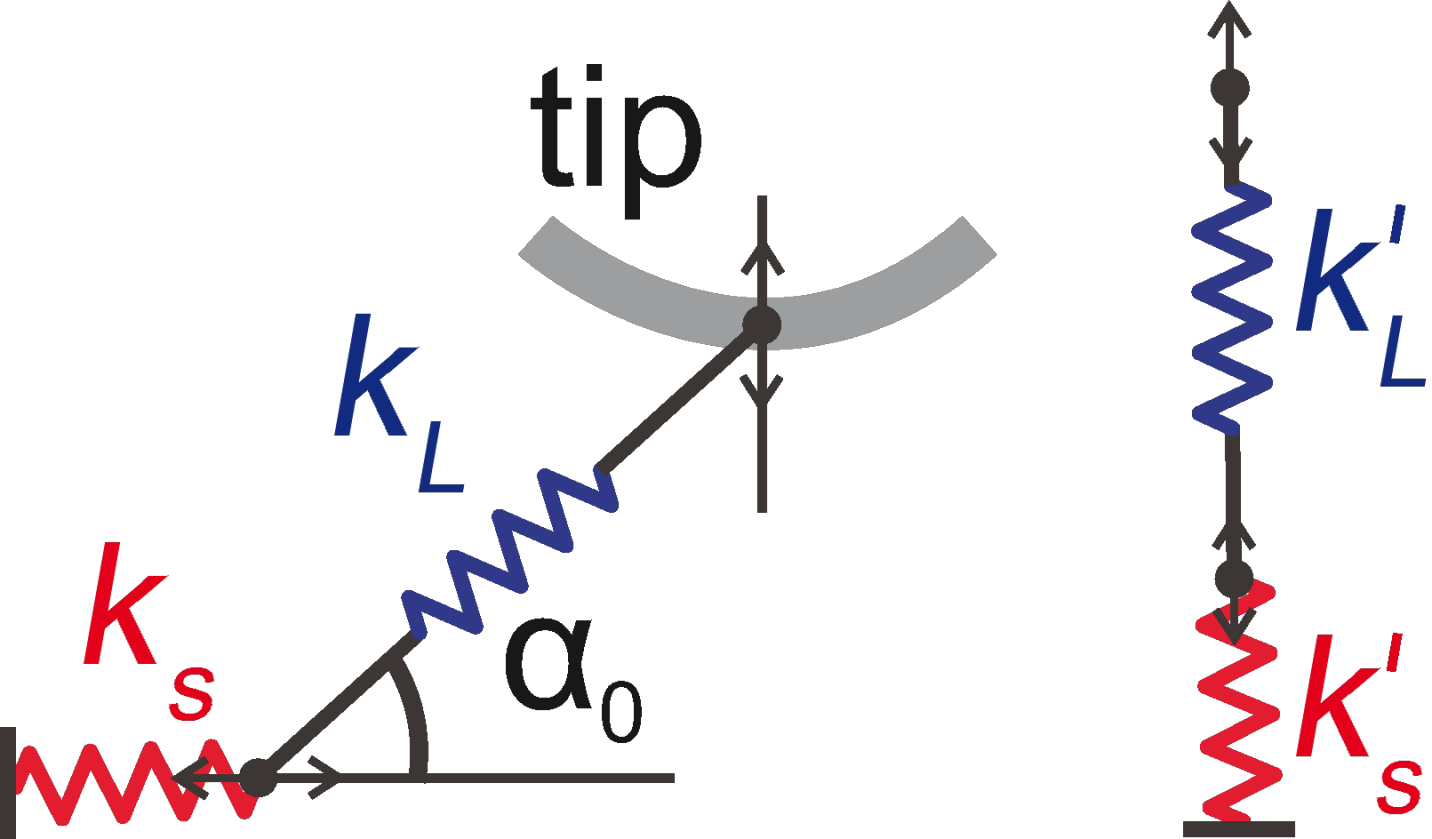
One-dimensional spring model of the manipulation process. Left: The molecule is represented by a spring with stiffness *k_L_*, the surface corrugation by a spring with stiffness *k_S_*. The molecule is tilted by an angle *α*_0_ with respect to the surface. Right: For simplification, the two springs are replaced by vertical effective springs with constants 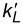 and 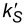. For more details, refer to the main text.

The way in which the surface corrugation affects *∂F_z_*/*∂z*(*z*) curves measured with NC-AFM can be understood by considering the model of an elastically stretchable (and compressible) rod lifted from a corrugated surface. The model consists of two connected springs, one of which mimics the elasticity of the rod, while the other accounts for the surface corrugation potential felt by the lower end of the rod (cf. Figure 3, left). In the model the motion of the lower end of the rod is confined to the surface. Since the sole purpose of this one-dimensional spring model is the analysis of the influence of surface corrugation on dynamic force measurements with the qPlus sensor, we assume that at each tilt angle *α*_0_ of the rod with respect to the surface plane both springs are fully relaxed (zero forces). This in fact corresponds to the situation of the molecule in the simulated junction when we allow lateral relaxation of the tip position at each step in *z*_tip_ (the vertical attractive forces in the simulated junction are of course non-zero, but since they do not play any role for understanding the influence of the surface corrugation on dynamic force measurements with the qPlus sensor, they are not included in the spring model of Figure 3, left).

The relevant quantity that we seek an expression for is the gradient of the force needed to move the upper end of the rod along the vertical, corresponding to the direction of oscillation of the qPlus sensor, evaluated at the relaxed position of the springs (equivalent to zero deflection *i*Δ*z* of the qPlus sensor). Initially assuming spring *S* to be infinitely stiff (*k_S_* → ∞), we find 
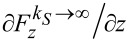
 = −*k_L_* sin^2^ *α*_0_ = 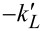 for spring *L*, and vice versa for an infinitely stiff spring *L* (*k_L_* → ∞) we find 
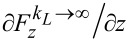
 = −*k_S_* tan^2^ *α*_0_ = 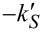 for spring *S*. A detailed derivation of these expressions can be found in Supporting Information File 1. We simplify the model by replacing *S* and *L* by two effective vertical springs, adding the spring constants derived in the two opposite limits, 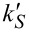 and 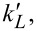 in series (Figure 3 right). In the present case this is a realistic approximation, because for most angles *α*_0_ the behavior of the model will be determined by the softer spring *S* (surface corrugation) and *L* can be considered as rigid, except for *α*_0_ close to 90°, where 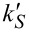 diverges while 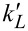 remains finite. The total spring constant of the system becomes

[2]
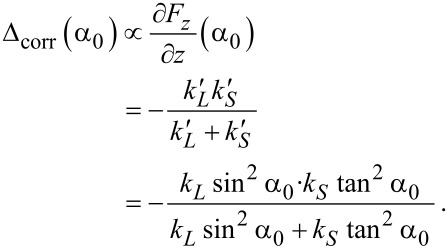


This expression reflects the basic properties of the correction term Δ_corr_(*α*_0_). Firstly, we find that Δ_corr_(*α*_0_) must always be negative (Δ*f* positive), and hence produce an additional repulsion after integration. Furthermore, Δ_corr_(*α*_0_) is zero for a molecule that is lying flat on the surface (*α*_0_ = 0) and it approaches the intrinsic stiffness of the molecule −*k_L_* for an upright standing orientation (*α*_0_ = 90°) where 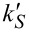 diverges. As the fit of Δ_corr_(*α*_0_) from the force field simulation in Figure 2b shows, the above analytic expression fully explains its qualitative behavior.

**Figure 4:**
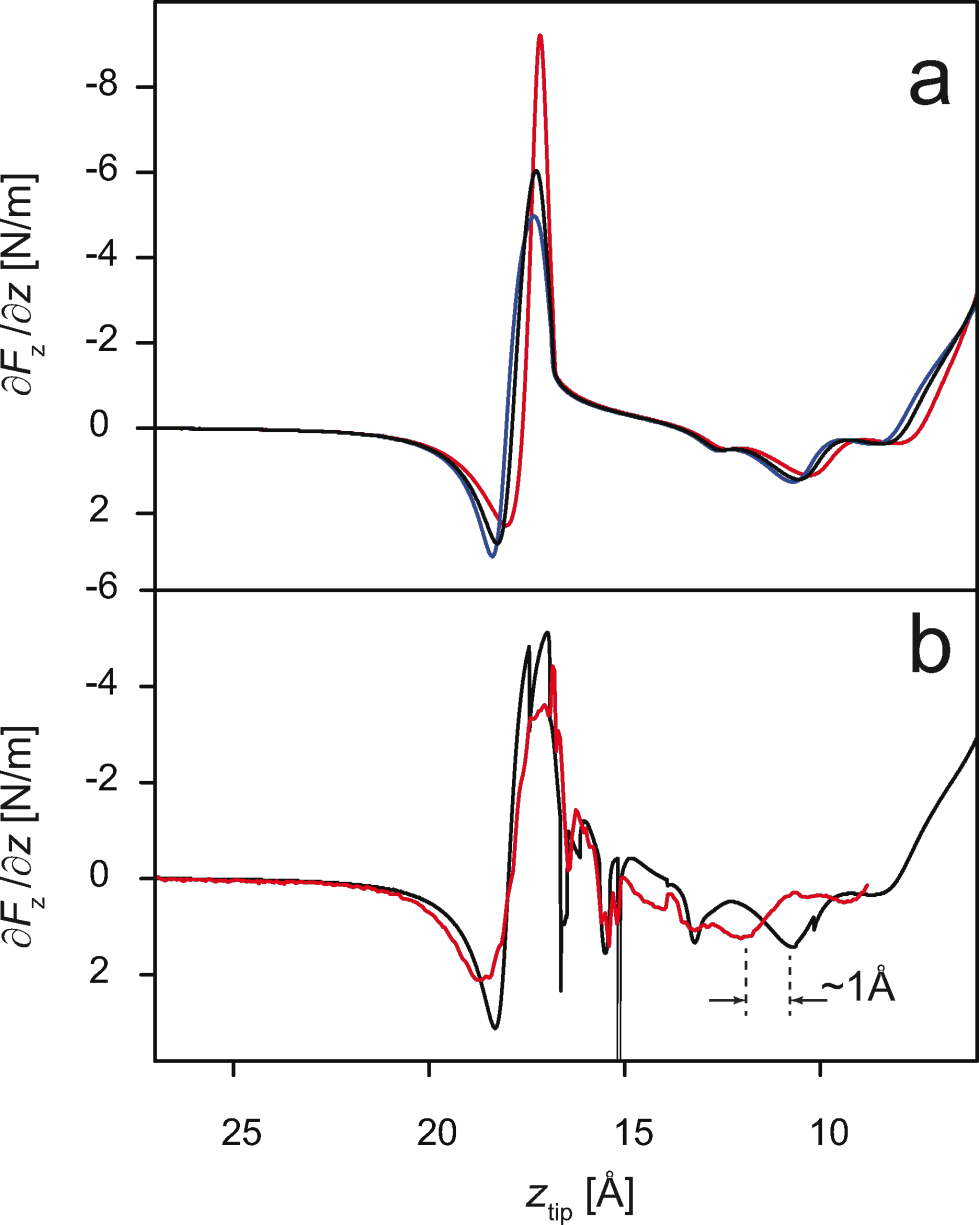
(a) Comparison between the *∂F_z_*/*∂z*(*z*) curves of PTCDA lifted from Au(111), obtained for different tip elastic constants. Simulations take oscillations of the qPlus sensor and the surface corrugation into account. Lateral forces are zeroed out (cf. text). Red corresponds to *k*_tip_ = ∞, blue to *k*_tip_ = 9 N/m, black to *k*_tip_ = 14 N/m. (b) Comparison between an individual experimental curve (red) taken from the series shown in Figure 1a and the simulation (black) obtained with oscillations of the qPlus sensor, tip flexibility, surface corrugation, and non-zero lateral forces acting during lifting. Sharp spikes in the black curve indicate sliding of the lower end of PTCDA across the corrugated potential of the surface.

Having studied the influence of the tip oscillation and the surface corrugation potential, we turn to the next simulation run. This time we additionally allow the tip to deform (in the vertical direction only) in the course of the lifting process. In Figure 4a we compare simulations performed for two different *k*_tip_ values that were chosen according to typical values reported for atomic nano-contacts [26]. Apparently, the softening of the tip has a strong influence on the shapes of the calculated *∂F_z_*/*∂z*(*z*_tip_) curves. This influence is strongest when the force pulling the tip towards the surface is large. The first of such instances can be observed in the *z*_tip_ range in which the naphthalene units of PTCDA are detached from the surface (8 Å ≤ *z*_tip_ ≤ 12 Å, cf. Figure 1b), while the second occurs during the final detachment of the molecule (17 Å ≤ *z*_tip_ ≤ 20 Å). In both cases the effect of the soft tip manifests itself as a partial decoupling between the position *z* of the tip (i.e., the read-out in the experiment of the position of the piezo-actuator to which the tip is attached) and the position *z*_tip_ of the microscopic tip apex, the latter determining the actual junction structure. As a result, each time the attractive force acting on the tip apex rises, the microscopic tip apex gets elongated and thus the features of the *∂F_z_*/*∂z*(*z*_tip_) curve are shifted to higher *z*_tip_ values. As soon as the attractive force decreases, the tip apex shrinks back, thus synchronizing the microscopic and the macroscopic *z*-scales again. Overall, allowing elastic tip deformations improves the agreement of the simulated curve with the experimental one, mostly by smearing out the sharp *∂F_z_*/*∂z*(*z*_tip_) peak.

Finally, we combine our findings regarding surface corrugation and tip stiffness to perform the most realistic simulation of our single-molecule manipulation experiments yet. As in the experiment, we use a strictly vertical tip trajectory that leads to a sliding motion of the lower end of the molecule across the surface prior to its detachment from the surface. The sliding motion manifests itself in the simulations as a series of spikes in *∂F_z_*/*∂z*(*z*_tip_) (cf. Figure 4b). Qualitatively, the spikes produced in the simulation look similar to the features observed in the individual *∂F_z_*/*∂z*(*z*) curves recorded in the experiment. Furthermore, both in the simulated and experimental curves the spike density on the *z*-axis increases as the molecule approaches the upright configuration. This is fully consistent with the assumption that the spikes are due to the lower end of the molecule sliding across the corrugation potential of the surface: Indeed, as the molecule stands-up the frequency of the sliding events per unit distance of the vertical tip retraction must increase. By the same token, it is very unlikely that the spikes result from structural changes in the tip. Such changes would be expected to occur mostly where the vertical force on the tip is strong (for *z*_tip_ < 12 Å and 16.5 Å < *z*_tip_ < 18 Å; see Figure 1c). However, this is not the region in which spikes are observed in the experiment. Lateral forces are not expected to play a role in deforming the tip. The simulation shows that due to the weak surface corrugation they are approximately ten times smaller than the vertical force.

Comparing the result of the final simulation run to the experiment, we note further that the overall fit quality is not perfect. In particular, the detachment of the naphthalene units in the simulation still happens 1 Å closer to the surface (compare Figure 1b to Figure 4b). To address the remaining discrepancies, it would be necessary to refine the parameter set describing the interaction of PTCDA with the Au(111) surface.

## Conclusion

In summary, we have simulated the lifting of a single PTCDA molecule from the surface using an extended force-field model that accounts for both surface corrugation and tip elasticity. Most importantly, the model also explicitly includes the finite oscillation amplitude of the qPlus tuning fork sensor. This has been achieved by the direct calculation of the qPlus oscillation frequency, solving the equation of motion of the tip within a full oscillation cycle. We have shown that the oscillation of the sensor together with the corrugation of the surface adds a positive frequency shift to the measurement that generates an apparent repulsive force. This contribution that we refer to as the correction term Δ_corr_ should get stronger with increasing corrugation. Therefore, we suggest that for strongly interacting surfaces its influence may dominate the measurement, in which case the measured force might seem repulsive during the whole molecular lifting process.

Finally, we have demonstrated that the scattering observed in the experiments is related to the sliding of the molecule across the surface that occurs in a certain *z*-range. Control over the sliding motion could be very difficult to achieve, since it requires control of the initial adsorption site and, in the best case, vanishing oscillation amplitude of the qPlus sensor. We thus conclude that for a fully controlled molecular lifting experiment it is desirable to lift molecules along trajectories that minimize the lateral forces in the junction.

## Supporting Information

File 1 Derivation of the two-spring model
