# Supplementary material for: The role of surface corrugation and tip oscillation in single-molecule manipulation with a non-contact atomic force microscope
Source: Beilstein J Nanotechnol. 2014 Feb 26;5:202–9. doi: 10.3762/bjnano.5.22 (PMC3943512; doi:10.3762/bjnano.5.22)
Supplement: File 1 — Derivation of the two-spring model [file Beilstein_J_Nanotechnol-05-202-s001.pdf]

Supporting Information

for

The role of surface corrugation and tip oscillation in single-molecule manipulation with a non-contact atomic force microscope

Christian Wagner*[1,2,3], Norman Fournier[2,3], F. Stefan Tautz[2,3] and Ruslan Temirov[2,3]

Address: [1]Leiden Institute of Physics, Universiteit Leiden, Niels Bohrweg 2, 2333 CA Leiden, The Netherlands; [2]Peter Grünberg Institut (PGI-3), Forschungszentrum Jülich, 52425 Jülich, Germany and [3]Jülich Aachen Research Alliance (JARA)-Fundamentals of Future Information Technology, 52425 Jülich, Germany

Email: Christian Wagner* - c.wagner@fz-juelich.de

* Corresponding author

Derivation of the two-spring model

Figure S1 shows the two-spring model employed in the paper. Point A ("tip") is constrained to move on the vertical axis (z-axis), while point B ("surface-bonded lower end of the molecule = carboxylic oxygen atom") is constrained to slide on the horizontal axis (x-axis). Point C is fixed. At a given angle α_0, springs S and L are relaxed, at lengths S_0 and L_0, respectively. In the course of the lifting experiment, the molecule (spring L) is lifted from $\alpha_0 = 0$ to $\alpha_0 = 90°$. For each α_0, the geometry of the model is adjusted such that both springs L and S are relaxed for $z = \sqrt{L_0^2 - S_0^2}$. At each α_0, the tip (point A) oscillates around the equilibrium z (qPlus sensor). If point A oscillates around its equilibrium position, point B will slide in the horizontal direction, and both springs L and S will be compressed or extended, depending on the direction of the displacement of point A.

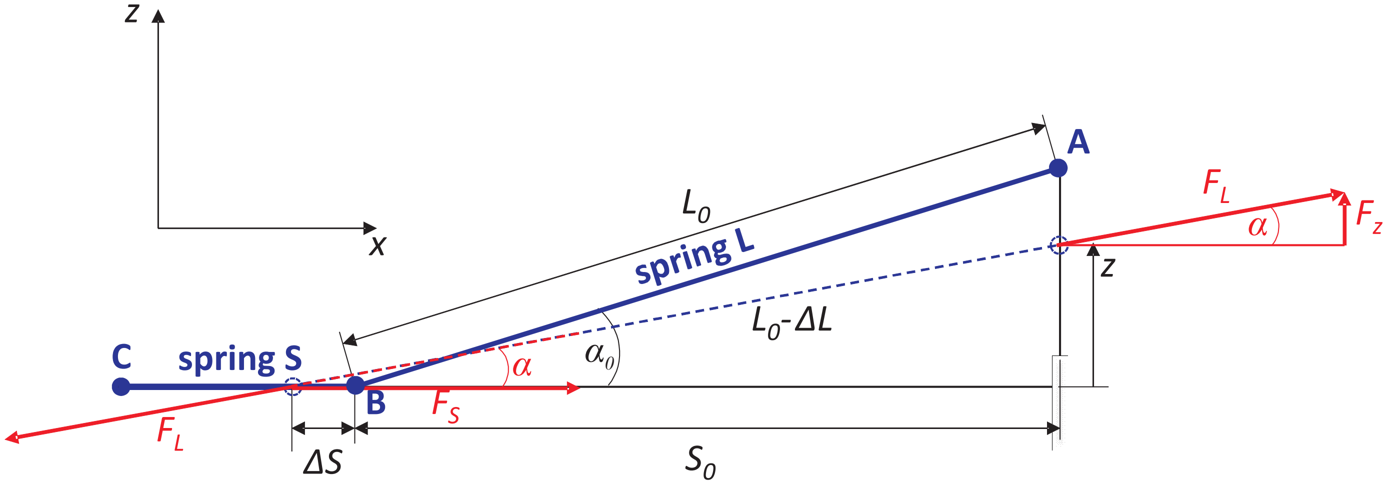

Figure S1: Sketch of the two-spring model employed in the paper.

We are interested in the vertical gradient of the vertical force F_z acting at point A around its equilibrium position, since this determines the frequency shift of the qPlus sensor in our experiment.

At point A, the following equation holds for the force F_z

$$F_z = -F_L \sin \alpha, \tag{1}$$

while at point B we have

$$F_S = -F_L \cos\alpha. \tag{2}$$

The sign convention for forces and displacements is illustrated in the figure. For the springs we have

$$F_S = k_S \Delta S \tag{3}$$

and

$$F_L = -k_L \Delta L. \tag{4}$$

Note that $k_L \gg k_S$. Finally, there are three geometrical relations, namely

$$\frac{S_0 + \Delta S}{L_0 - \Delta L} = \cos\alpha, \tag{5}$$

$$\frac{z}{L_0 - \Delta L} = \sin\alpha, \tag{6}$$

$$(S_0 + \Delta S)^2 + z^2 = (L_0 - \Delta L)^2. \tag{7}$$

Eliminating F_S, F_L, α, ΔS and ΔL, we obtain from Equations 1–7

$$\frac{L_0^2 z^2}{\left(z + \frac{F_z}{k_L}\right)^2} = z^2 + S_0^2 \left(1 + \frac{F_z}{k_S z - F_z}\right)^2, \tag{8}$$

which yields $F_z(z)$ as a function of the parameters L_0, S_0, k_L, and k_S. In principle, Equation 8 allows the determination of $\partial F_z/\partial z$. However, we will derive a simplified expression for $\partial F_z/\partial z$ by analysing two limiting cases of Equation 8.

Equation 8 can be discussed in two limiting cases:

- $k_L \to \infty$, i.e. the molecule is modeled as a rigid rod. In that case, we can simplify Equation 8,

solve for F_z and find

$$F_z^{k_L\to\infty} = -k_S z\left[\frac{S_0}{\sqrt{L_0^2 - z^2}} - 1\right] = -k_S z\left[\frac{\cos\alpha_0}{\sqrt{1 - z^2/L_0^2}} - 1\right], \tag{9}$$

with $\cos\alpha_0 = S_0/L_0$. For horizontal L and S springs, i.e. $z = 0$, or for relaxed springs, i.e. $z = \sqrt{L_0^2 - S_0^2}$ at arbitrary α_0, the equation yields $F_z^{k_L\to\infty} = 0$, as it must. Furthermore, $F_z^{k_L\to\infty} \geq 0$ for $z \leq \sqrt{L_0^2 - S_0^2}$, $F_z^{k_L\to\infty} < 0$ for $z > \sqrt{L_0^2 - S_0^2}$, and $F_z^{k_L\to\infty} \to -\infty$ for $z \to L_0$. From Equation 9 the force gradient becomes

$$\frac{\partial F_z^{k_L\to\infty}}{\partial z} = -k_S\left[\frac{S_0 z^2}{(L_0^2 - z^2)^{3/2}} + \frac{S_0}{(L_0^2 - z^2)^{1/2}} - 1\right] \tag{10}$$

$$= -k_S\left[\frac{z^2\cos\alpha_0}{L_0^2(1 - z^2/L_0^2)^{3/2}} + \frac{\cos\alpha_0}{(1 - z^2/L_0^2)^{1/2}} - 1\right] \tag{11}$$

and, at the relaxed position of the springs $z = \sqrt{L_0^2 - S_0^2}$,

$$\left.\frac{\partial F_z^{k_L\to\infty}}{\partial z}\right|_{z=\sqrt{L_0^2 - S_0^2}} = -k_S\tan^2\alpha_0 \equiv -k_S' \tag{12}$$

For $\alpha_0 = 0$, the force gradient vanishes, just as the force itself, as is to be expected. As α_0 increases, the influence of the lateral corrugation on the force gradient and therefore the measured frequency shift increases. At $\alpha_0 = 90°$, the force gradient becomes infinite because of the infinite stiffness of the rod.

- $k_S \to \infty$, i.e. an infinitely strong lateral surface corrugation. In that case, we find from Equation 8

$$F_z^{k_S\to\infty} = -k_L z\left[1 - \frac{L_0}{\sqrt{S_0^2 + z^2}}\right] = -k_L z\left[1 - \frac{1}{\cos\alpha_0\sqrt{1 + z^2/S_0^2}}\right], \tag{13}$$

with $\cos\alpha_0 = S_0/L_0$. For horizontal L and S springs, i.e. $z = 0$, or for relaxed springs, i.e. $z = \sqrt{L_0^2 - S_0^2}$, at arbitrary α_0, the equation yields $F_z^{k_S\to\infty} = 0$, as it must. Furthermore, $F_z^{k_S\to\infty} \geq 0$

for $z \leq \sqrt{L_0^2 - S_0^2}$, $F_z^{k_S \to \infty} < 0$ for $z > \sqrt{L_0^2 - S_0^2}$, and $F_z^{k_S \to \infty} \to -zk_L \to -\infty$ for $z \to \infty$. From Equation 13 the force gradient becomes

$$\begin{aligned} \frac{\partial F_z^{k_S \to \infty}}{\partial z} &= -k_L \left[1 + \frac{L_0 z^2}{(S_0^2 + z^2)^{3/2}} - \frac{L_0}{(S_0^2 + z^2)^{1/2}} \right] && (14) \\ &= -k_L \left[1 + \frac{z^2}{S_0^2 \cos\alpha_0 (1 + z^2/S_0^2)^{3/2}} - \frac{1}{\cos\alpha_0 (1 + z^2/S_0^2)^{1/2}} \right] && (15) \end{aligned}$$

which yields

$$\left. \frac{\partial F_z^{k_S \to \infty}}{\partial z} \right|_{z = \sqrt{L_0^2 - S_0^2}} = -k_L \sin^2\alpha_0 \equiv -k'_L \qquad (16)$$

For $\alpha_0 = 0$, the force gradient vanishes, just as the force itself, as is to be expected. As α_0 increases, the force gradient and therefore the measured frequency shift increases, because varying z now requires increasing length changes of the rod. At $\alpha_0 = 90°$, the force gradient becomes $-k_L$, i.e. equal to the stiffness of the rod.

In our experiment, we expect the compressive and tensile stiffness of the molecule to be much larger than the lateral corrugation. Therefore, for most angles α_0 the limit $k_L \to \infty$ is a realistic model of our experiment. Only for angles α_0 which are close to 90° we expect the finite stiffness of the molecule (spring L) to become relevant, because then the effective spring constant k'_S (Equation 12) diverges, and thus the intrinsic stiffness of L becomes smaller than k'_S. Hence, the total force gradient $\partial F_z / \partial z$ can be represented by two effective springs k'_S and k'_L added in series. At most angles, spring k'_S is the softer (and hence the only relevant) one. Only close to the upright configuration does k'_L become softer and relevant. The total force gradient of our model is thus given by

$$\left. \partial F_z / \partial z \right|_{z = \sqrt{L_0^2 - S_0^2}} = -\left(\frac{1}{k'_L} + \frac{1}{k'_S} \right)^{-1} = -\frac{k'_L k'_S}{k'_L + k'_S} = -\frac{k_L \sin^2\alpha_0 \cdot k_S \tan^2\alpha_0}{k_L \sin^2\alpha_0 + k_S \tan^2\alpha_0} \qquad (17)$$

The implications of this expression for the experiment is discussed in the paper.
